# Association of Myeloid Cells of Triggering Receptor-1 with Left Ventricular Systolic Dysfunction in BALB/c Mice with Sepsis

**DOI:** 10.1155/2014/391492

**Published:** 2014-05-15

**Authors:** Gaosheng Zhou, Lijun Ye, Liang Zhang, Liangqing Zhang, Yuanli Zhang, Liehua Deng, Huaguo Yao

**Affiliations:** Department of Critical Care Medicine, Affiliated Hospital of Guangdong Medical College, No. 57 Southern Renmin Avenue, Zhanjiang, Guangdong 524023, China

## Abstract

*Objective*. To investigate the correlation between TREM-1 and LPS-induced left ventricular systolic dysfunction in BALB/c mice. * Methods*. Male BALB/c mice were randomly divided into 3 groups: LPS, LPS/TREM-1, and control groups which were injected intraperitoneally with 25 mg/kg LPS, 5 **μ**g TREM-1mAb 1 h after LPS challenge, and sterilized normal saline, respectively. Left ventricular systolic function was monitored by echocardiography at 6 h, 12 h, and 24 h. Meanwhile, TNF-**α**, IL-1**β**, and sTREM-1 in serum and myocardium were determined by ELISA or real-time PCR; at last left ventricles were taken for light microscopy examination. * Results*. FS and EF in LPS/mAbTREM-1 group, significantly declined compared with LPS and control group at 12 h, were accompanied with a markedly increase in serum IL-1**β** (at 6 h) and sTREM-1 (at 12 h and 24 h) expression. Myocardium TNF-**α** (at 6 h and 24 h) and sTREM-1 (at 6 h) were significantly higher in LPS/mAbTrem-1-treated mice than in time-matched LPS-treated mice; meanwhile myocardium TNF-**α** mRNA were markedly increased in comparison with LPS-treated or saline-treated mice at 24 h. Besides, mAbTREM-1 aggravated LPS-induced myocardial damage was observed. * Conclusions*. Our results suggest that TREM-1 is significantly associated with LPS-induced left ventricular systolic dysfunction in BALB/c mice.

## 1. Introduction

The presence of cardiovascular dysfunction is an early and fatal complication for patients with sepsis or septic shock, with the mortality rate of 70% to 90% compared with 20% in septic patients without cardiovascular impairment [1, 2]. And approximately 64% of patients with severe sepsis or septic shock presented with myocardial depression including left ventricular diastolic and systolic dysfunction and right ventricular dysfunction [3].

Cardiovascular dysfunction during sepsis is probably multifactorial, and inflammatory cytokines have been shown to be clearly associated with it, but the precise molecular mechanisms remain unclear [4]. Furthermore, the previous study showed that proinflammatory mediators, such as tumor necrosis factor-*α* (TNF-*α*) and interleukin-1*β* (IL-1*β*), played a critical part in myocardial dysfunction during sepsis [5].

Triggering receptor 1 expressed on myeloid cells (TREM-1), a newly discovered receptor of the immunoglobulin superfamily, activates neutrophils and monocytes/macrophages through an associated signal transduction molecule DAP12 [6]. Engagement of TREM-1 has been reported to trigger the synthesis of proinflammatory cytokines such as TNF-*α* and IL-1*β* in the presence of LPS, leading to systemic inflammatory response syndrome [7–9]. As described above, the release of proinflammatory cytokines (TNF-*α* and IL-1*β*) leads to cardiac depression or dysfunction following sepsis. Hence, we hypothesize that TREM-1 could play an important role in lipopolysaccharide (LPS)-induced cardiac dysfunction. Furthermore, our previous observation found that serum sTREM-1 level was significantly associated with cardiac index (CI), global ejection fraction (GEF), and left ventricular systolic index (dp/dt max) in patients with severe sepsis [10]. Thus, the purpose of this study was to investigate the association of myeloid cells of triggering teceptor-1 with left ventricular systolic dysfunction in BALB/c mice with sepsis.

## 2. Materials and Methods

### 2.1. Materials

LPS (*Escherichia coli* 055:B5) was purchased from Sigma Chemical Co. (St. Louis, MO, USA); mouse TREM-1 monoclonal antibody (mAb) was purchased from R&D Systems Inc. (Minneapolis, MN, USA). LPS was dissolved in sterile saline to a concentration of 1.0 mg/mL, and TREM-1 mAb was reconstituted at 0.5 mg/mL in sterile PBS.

#### 2.1.1. Ethics Statement

All experiments were conducted in accordance with the guidelines for the care and use of laboratory animals by the Experimental Animal Care and Ethical Committee of Nanfang Medical University.

#### 2.1.2. Experimental Animals and Experimental Design

Ninety male BALB/c mice (age: 7 to 9 weeks and weight: 20 to 25 g) were purchased from Nanfang Medical University Experimental Animal Center; they were randomly divided into 3 groups, each of 30 animals. LPS, LPS/TREM-1, and control groups were injected intraperitoneally with 25 mg/kg LPS, 5 *μ*g TREM-1 mAb 1 h after LPS challenge, and sterilized normal saline, respectively. Each group was further divided into 3 subgroups according to different time-points, that is, 6 h, 12 h, and 24 h. The left ventricular systolic function was evaluated in 5 mice of each subgroup, and the myocardium and blood sample were collected in the remainder at 3 time-points. The doses of LPS and TREM-1 mAb were utilized based on previous study [11]. All animals were housed in plastic cages (5 mice per cage) under standard laboratory conditions and mice were maintained on a 12 h light/dark cycle with free access to regular rodent chow and tap water. The experimental animal models were redone to get the blood and tissue specimens or echocardiographic assessment if the mice died before/at 3 time-points.

### 2.2. Echocardiographic Assessment

According to the report of Ha et al. [12], 2D guided M-mode echocardiography with 15L8 probe (Contrast Pulse Sequencing, Sequoia 512, Siemens, Germany) was used to assess mouse left ventricular systolic function at 3 time-points. M-mode tracings were used to measure the left ventricular end-systolic diameter (LVESd) and left ventricular end-diastolic diameter (LVEDd) from 5 consecutive cardiac cycles. Left ventricular fractional shortening (FS) index and ejection fraction (EF) index were calculated as described previously [13]. All measurements were made by the same observer who was blinded with the experimental grouping.

### 2.3. ELISA

Hearts were harvested at specific times, and homogenates were prepared as previously described [14]. Serum and myocardial concentrations of TNF-*α*, IL-1*β*, and sTREM-1 in all mice at 3 time-points were determined using ELISA kits (JiangLai, Shanghai, China) according to the manufacturer's protocols.

### 2.4. Quantitative Real-Time RT-PCR

Total RNA was extracted from frozen cardiac tissues obtained from all mice at 3 time-points, using RNAzol solution (Biogenesis) according to the manufacturer's instructions. Myocardial TNF-*α*, IL-1*β*, and sTREM-1 expression were measured by quantitative real-time RT-PCR with iCycler iQ real-time PCR system (Bio-Rad). Primer pairs used were as follows:

 TNF-*α*: forward 5′-CCCCAAAGGGATGAGAAGTTC-3′,
 reverse 5′-GGCTTGTCACTCGAATTTTGAGA-3′;
 IL-1*β*: forward 5′-CCAAAAGATGAAGGGCTGCTT-3′,
 reverse 5′-GAAAAGAAGGTGCTCATGTCCTC-3′;
 sTREM-1: forward 5′-GACGGGAAGGAACCCTTGA-3′,
 reverse 5′-CATGGCCTCACTAGGGTCATGT-3′;
 GAPDH: forward 5′-CGTGTTCCTACCCCCAATGT-3′,
 reverse 5′-TGTCATCATACTTGGCAGGTTTCT-3′.

Each sample was tested in triplicate for each gene; relative levels of gene expression were normalized to GAPDH expression levels.

### 2.5. Histopathological Examination

Paraffin embedded heart tissues were sectioned and stained with hematoxylin and eosin as previously described [15]. Histopathological examination was observed by an expert who was unaware of the experimental allocation of the animals.

### 2.6. Statistical Analysis

Data are reported as means and standard error of the mean (SEM). Significant differences among three groups were determined by one-way analysis of variance. Statistical analysis was performed using GraphPad Prism 5.0 (GraphPad Software, San Diego, California, USA). A *P* value of less than 0.05 was considered statistically significant.

## 3. Results

### 3.1. Clinical Manifestations in Mice after LPS Challenge

To study the potential function of TREM-1 in the pathogenesis of myocardial dysfunction in septic mice, we observed that LPS/mAbTREM-1-treated and LPS-treated mice showed signs of sepsis such as absence of grooming activities with resulting ruffled fur, olfactory discharge, and diarrhea, no oral uptake of food or water, and lethargy compared with saline-treated controls after LPS administration at different times. Besides, few of mice in LPS and LPS/mAbTREM-1 group died within the first 24 h.

### 3.2. mAbTREM-1 Exacerbates LPS-Induced Left Ventricular Systolic Dysfunction

To investigate the effect of TREM-1 in the pathogenesis of left ventricular systolic dysfunction in septic mice, we employed echocardiography as a noninvasive approach to assess left ventricular systolic function at 6 h, 12 h, and 24 h in 3 groups.

**Figure 1:**
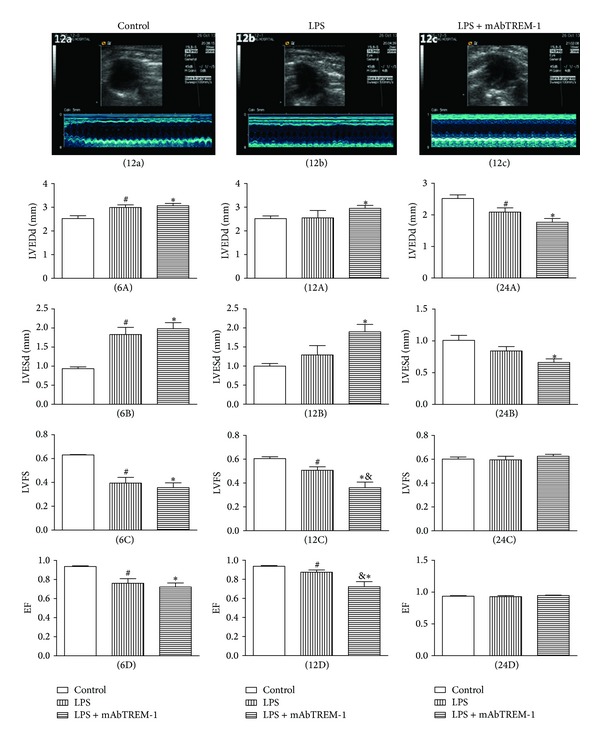
mAbTREM-1 deteriorates left ventricular systolic function in mice following LPS treatment. (12a), (12b), and (12c) represent M-mode echocardiograms among 3 groups at 12 h, respectively. Bar graphs demonstrating differences in echocardiographic left ventricular parameters (LVEDd, LVESd, FS, EF) at 6 h ((6A)–(6D)), 12 h ((12A)–(12D)), and 24 h ((24A)–(24D)) among 3 groups as described in Materials and Methods; LVESd: left ventricular end-systolic diameter, LVEDd: left ventricular end-diastolic diameter, FS: fractional shortening (%), and EF: ejection fraction (%); data are expressed as MEAN ± SEM; ^#^ *P* < 0.05 compared with saline-treated mice, *P < 0.05 compared with saline-treated mice, and ^&^ *P* < 0.05 compared with LPS-treated mice.

FS and EF were severely decreased in both LPS-treated and LPS/mAbTREM-1-treated mice, when compared to the control group. LPS-induced left ventricular systolic dysfunction was evident at 6 h, pronounced at 12 h, and gradually returned to near baseline values at 24 h after LPS challenge. As expected, 12 h after LPS/mAbTREM-1 challenge, the mice had a lower FS and EF than did LPS-treated mice (FS: 50.7% versus 36.1% at 12 h and EF: 87.5% versus 72.3%, both *P* < 0.05, Figure 1 (12C) and (12D)). However, there was no significant difference of both FS and EF between LPS-treated and LPS/mAbTREM-1-treated mice at 6 h and 24 h (Figure 1 (6C), (24C), (6D), and (24D), *P* > 0.05). Interestingly, no significant differences were observed on LVESd and LVEDd between LPS and LPS/mAbTREM-1 groups at 3 time-points (Figure 1 (6A)–(24A) and (6B)–(24B), *P* > 0.05).

### 3.3. Effect of mAbTREM-1 on the Levels of Proinflammatory Cytokines in Myocardium and Serum during Sepsis

To investigate the effect of TREM-1 pretreatment on production of cytokines in septic mice induced by LPS, the levels of TNF-*α*, IL-1*β*, and sTREM-1 in serum and myocardium were determined at protein levels by ELISA at 3 time-points among 3 groups.

**Figure 2:**
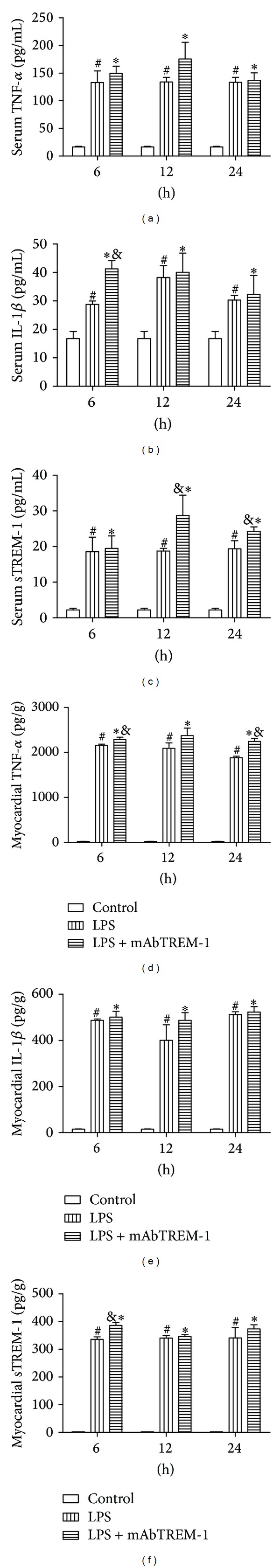
Serum TNF-*α* (a), IL-1*β* (b), sTREM-1 (c) and myocardium TNF-*α* (d), IL-1*β* (e), and sTREM-1 (f) levels at 6 h, 12 h, and 24 h among three groups as described in Materials and Methods; data are expressed as MEAN ± SEM; ^#^ *P* < 0.05 compared with saline-treated mice, *P < 0.05 compared with saline-treated mice, and ^&^ *P* < 0.05 compared with LPS-treated mice.

As shown in Figure 2, the basic serum and myocardial TNF-*α*, IL-1*β*, and sTREM-1 levels in control mice were lower detectable; normal saline did not influence these values. The levels of TNF-*α*, IL-1*β*, and sTREM-1 were significantly increased in myocardium and serum of both LPS/mAbTREM-1-treated and LPS-treated mice compared with saline-treated mice at 3 time-points, respectively (Figures 2(a)–2(f), *P* < 0.05).

Myocardium TNF-*α* expression was markedly increased in LPS/mAbTREM-1-treated mice in comparison with LPS-treated at 6 h and 24 h (Figure 2(a), *P* < 0.05). However, no significant differences were found in serum TNF-*α* expression between LPS and LPS/mAbTREM-1 mice at 3 time-points.

Serum IL-1*β* expression in LPS/mAbTREM-1-treated mice exhibited marked increases compared with the LPS-treated mice at 6 h (Figure 2(b), *P* < 0.05) and indicated that administration of mAbTREM-1 to endotoxemic mice markedly increased in IL-1*β* expression of serum. But there were no significant differences in myocardium IL-1*β* expression between LPS-treated and LPS/mAbTREM-1-treated mice at 3 time-points.

Serum sTREM-1 expression in LPS/mAbTREM-1-treated mice was significantly increased compared with LPS-treated mice at 12 h and 24 h (Figure 2(c), *P* < 0.05), and the same trend was observed in myocardium at 6 h between LPS/mAbTREM-1-treated and LPS-treated mice (Figure 2(e), *P* < 0.05).

### 3.4. Effect of mAbTREM-1 on the Transcript Levels of Proinflammatory Cytokines in the Myocardium with Septic Mice

**Figure 3:**
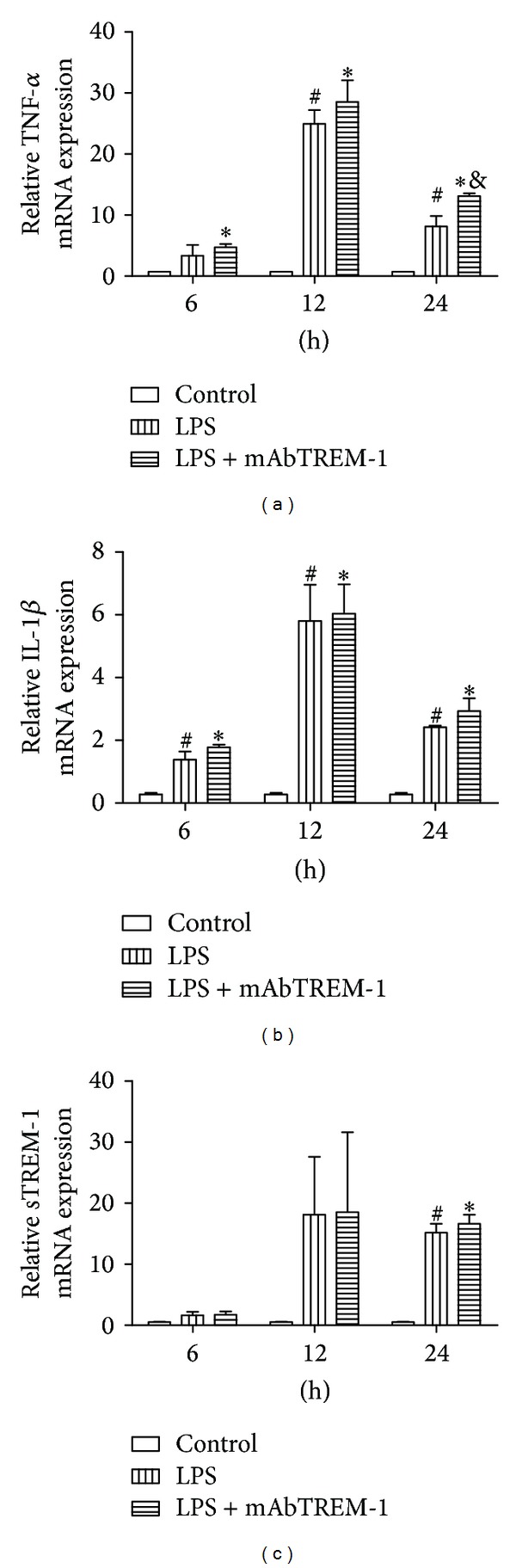
Myocardial TNF-*α* (a), IL-1*β* (b), and sTREM-1 (c) mRNA expression were assayed by quantitative real-time PCR among 3 groups at 6 h, 12 h, and 24 h as described in Materials and Methods; data are expressed as MEAN ± SEM; ^#^ *P* < 0.05 compared with saline-treated mice, *P < 0.05 compared with saline-treated mice, and ^&^ *P* < 0.05 compared with LPS-treated mice.

As shown in Figure 3, myocardial TNF-*α*, IL-1*β*, and sTREM-1 expression among 3 groups at 3 time-points were examined at transcript levels by real-time PCR.

The levels of myocardial TNF-*α*, IL-1*β*, and sTREM-1 mRNA were increased between LPS/mAbTREM-1-treated and LPS-treated mice after LPS exposure compared to those of saline-treated controls. Cardiac TNF-*α* mRNA expressions in LPS-treated and LPS/mAbTREM-1-treated mice were markedly higher than in saline-treated mice at 12 h and 24 h. And the same trend on IL-1*β* mRNA expression was found at 3 time-points between the two groups. Similarly, sTREM-1 mRNA expression was significantly greater in LPS/mAbTREM-1-treated mice and LPS-treated mice than in saline-treated mice at 24 h, respectively (Figures 3(a), 3(b), and 3(c), *P* < 0.05).

Myocardial TNF-*α* mRNA expression in LPS/mAbTREM-1-treated mice was markedly higher than in LPS-treated mice at 24 h (Figure 3(a), *P* < 0.05). However, there were no significant differences at the other 2 time-points (Figure 3(a), *P* > 0.05).

Although the levels of myocardial IL-1*β* and sTREM-1 mRNA in LPS/mAbTREM-1-injected were higher than in LPS-treated mice at 6 h, 12 h, and 24 h, there were no statistical differences (Figures 3(b) and 3(c), *P* > 0.05).

### 3.5. mAbTREM-1 Aggravates LPS-Induced Myocardial Damage

To evaluate the role of TREM-1 in the pathogenesis of myocardial impairment during sepsis, histological analysis was performed at 3 time-points.

**Figure 4:**
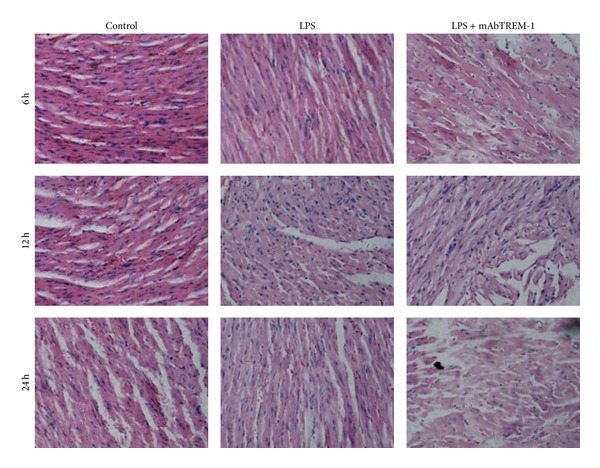
Morphological analysis of myocardial damage among 3 groups at 6 h, 12 h, and 24 h as described in Materials and Methods. Magnification ×400, extensive interstitial edema, infiltration of inflammatory cells, and hemorrhage in LPS-treated mice. Note that mabTREM-1 induced myocardium damage is markedly increased in LPS/mAbTREM-1-treated mice compared with saline-treated mice or LPS-treated mice.

Morphological examination displayed no significant difference in heart tissues of control group mice under light microscopy levels at 3 time-points. Hearts from LPS-treated mice displayed a mild feature of myocardial damage to some degree, including irregular arrangement and interstitial edema, infiltration of inflammatory cells, and slight hemorrhage at 3 time-points. Moreover, hearts from mice pretreatment with LPS/mAbTREM-1 also displayed myocardial damage to a certain extent at 6 h, 12 h, and 24 h. As expected, compared with these changes found in time-matched LPS mice, these lesions were considerably more severe in LPS/mAbTREM-1-treated mice (Figure 4).

## 4. Discussion

### 4.1. Cardiac Dysfunction Is One of the Major Complications of LPS-Induced Mice

Sepsis is the most important cause of morbidity and mortality in intensive care units (ICUs), and myocardial dysfunction frequently accompanies severe sepsis and septic shock [1, 2]. The previous study has showed that myocardial depression in septic shock has been well characterized in both spontaneous human septic shock and in experimental animal models of septic shock [16–18]. Similarly, this study indicated that left ventricular systolic dysfunction was observed in septic mice. We also observed that LPS treatment caused significant decrease of both EF and FS when compared with saline-treated mice at 6 h and 12 h.

### 4.2. Proinflammatory Mediators Play a Critical Part in Left Ventricular Systolic Dysfunction with Sepsis

Although the pathophysiology responsible for LPS-induced myocardial dysfunction remains unclear, several studies have shown that the large release of inflammatory mediators was an important factor [19].

#### 4.2.1. Inflammatory Cytokines Expression in Serum

We observed that serum TNF-*α*, IL-1*β*, and sTREM-1 production was markedly increased in mice pretreatment with LPS which accompanied a significant decrease of both left ventricular EF and FS compared with saline-treated mice at 6 h and 12 h. Furthermore, the previous experiments have showed hemodynamic changes and left ventricular systolic and diastolic dysfunction in mice after TNF-*α* or IL-1*β* administration [15]. Besides, inhibition of TNF-*α* or IL-1*β* can mitigate multiple organ dysfunctions and improve survival in septic mice [20–22]. Thus, it is suggested that TNF-*α*, IL-1*β*, and sTREM-1 in serum played an important part in LPS-induced left ventricular systolic dysfunction.

#### 4.2.2. Inflammatory Cytokines Expression in the Heart

The previous experiments showed that TNF-*α* and IL-1*β* were important inflammatory cytokines during sepsis, which were mainly produced by activated macrophages and cardiomyocytes [19]. As shown in Figures 1 and 3, the data indicating that LPS treatment caused marked increase of both myocardial TNF-*α*, IL-1*β*, and sTREM-1 protein and gene expression were paralleled by significant decrease of both EF and FS when compared with saline-treated mice at same time-points.

As described above, LPS treatment did cause significantly decrease of both EF and FS in comparison with the saline-treated mice, the inflammatory cytokines in serum and myocardium expression were markedly increased at the same time. Hence, we concluded that inflammatory cytokines played a critical part in left ventricular systolic dysfunction during sepsis. Due to various metabolic cycles, the difference of inflammatory cytokines between protein and gene expression appeared at different time-points.

### 4.3. TREM-1 as an Amplifier of the Inflammatory Response in Sepsis

TREM-1 is a newly discovered cell receptor, expressed on the surface of neutrophil and monocyte/macrophage, and can activate inflammation through TREM-1/DAP12 inflammatory signaling pathway [6–8]. As observed in this study, serum IL-1*β* (6 h) and sTREM-1 (12 h and 24 h) expression were significantly higher in LPS/mAbTrem-1-treated mice than in time-matched LPS-treated mice; meanwhile, we also observed the same trend on TNF-*α* (6 h and 24 h) and sTREM-1 (6 h) in myocardium between the 2 groups. TREM-1mAb that amplified the inflammation during sepsis was confirmed again in our study. On the other hand, the previous study indicated that blocking of TREM-1 can protect mice against LPS- or CLP-induced sepsis by mTREM-1/IgGl or LPl7 [23]. Thus, that means that TREM-1mAb did amplify the inflammation during sepsis.

Moreover, the mechanisms of LPS and TREM-1 signaling pathway reported that LPS binds to an acute-phase protein, LPS-binding protein (LBP), and activates CD14 on cell surfaces [24, 25]. Subsequently, a complex is formed among LPS/LBP, CD14, and a secreted protein (MD2), with toll-like receptor (TLR)-4, one of the recently described family of toll-like receptors [26, 27]. Next, expression of TREM-1 may be under the control of nuclear factor-*κ*B, with engagement of TREM-1 during sepsis possibly leading to activation of several transcription complexes that synergize with NF-*κ*B in order to elicit transcription of proinflammatory genes [28]. At last, large amounts of inflammatory cytokines release from activated inflammatory cells result in inflammation. Obviously, the similar reaction occurred in myocardium and serum during sepsis as this study.

### 4.4. TREM-1 as an Amplifier of the Inflammatory Response Plays a Pivotal Role in the Pathogenesis of LPS-Induced Left Ventricular Systolic Dysfunction

As our data indicated, mAbTREM-1 treatment indeed caused marked decrease of both EF and FS when compared with the LPS-treated mice; the same trend on serum/myocardium proinflammatory cytokines expression was observed at the same time. As mentioned above, mAb TREM-1 stimulates the expression of large amounts of TREM-1 during endotoxemia. Subsequently, TREM-1 can activate a cascade of proinflammatory cytokines in serum or myocardium (such as TNF-*α*, IL-1*β*, or sTREM-1), which appears to be a mediator of cytokine-induced left ventricular systolic dysfunction (FS and EF). Besides, we have previously reported the close relationship between cardiac dysfunction and serum sTREM-1 [10]. Furthermore, TREM-1 that aggravated LPS-induced myocardial damage was observed by histopathological examination. Thus, TREM-1 as an amplifier of the inflammatory response played a pivotal role in the pathogenesis of LPS-induced left ventricular systolic dysfunction.

Taken together, our present study demonstrated for the first time the association of TREM-1 with myocardial function in septic mice; as described above, TREM-1 had indeed proinflammatory properties and played an important role in the pathogenesis of LPS-induced left ventricular systolic dysfunction.

Limitation of the present study: our work is focused on the characteristics of TREM-1 in LPS-induced left ventricular systolic dysfunction in vivo. To understand the mechanism of TREM-1 in LPS-induced cardiac dysfunction, further studies with isolated myocardium from TREM-1^−/−^ and wild mice under LPS stimulation are needed, which may offer direct evidence for the effects of TREM-1 under LPS. That is the next work which we will do.

## 5. Conclusions

In summary, our results suggest that TREM-1 is significantly associated with LPS-induced left ventricular systolic dysfunction in BALB/c mice. The overexpression of TREM-1 plays an important role in the pathogenesis of LPS-induced left ventricular systolic dysfunction in mice. Thus, TREM-1 might be a therapeutic target for the treatment of cardiovascular dysfunction in sepsis.

## References

[1] Bulmer BJ (2011). Cardiovascular dysfunction in sepsis and citical illness. *Veterinary Clinics of North America: Small Animal Practice*.

[2] Parrillo JE, Parker MM, Natanson C (1990). Septic shock in humans. Advances in the understanding of pathogenesis, cardiovascular dysfunction, and therapy. *Annals of Internal Medicine*.

[3] Pulido JN, Afessa B, Masaki M (2012). Clinical spectrum, frequency, and significance of myocardial dysfunction in severe sepsis and septic shock. *Mayo Clinic Proceedings*.

[4] Merx MW, Weber C (2007). Sepsis and the heart. *Circulation*.

[5] Court O, Kumar A, Parrillo JE, Kumar A (2002). Clinical review: myocardial depression in sepsis and septic shock. *Critical Care*.

[6] Tessarz AS, Cerwenka A (2008). The TREM-1/DAP12 pathway. *Immunology Letters*.

[7] Colonna M, Facchetti F (2003). TREM-1 (triggering receptor expressed on myeloid cells): a new player in acute inflammatory responses. *Journal of Infectious Diseases*.

[8] Bouchon A, Dietrich J, Colonna M (2000). Cutting edge: inflammatory responses can be triggered by TREM-1, a novel receptor expressed on neutrophils and monocytes. *Journal of Immunology*.

[9] Bouchon A, Facchetti F, Weigand MA, Colonna M (2001). TREM-1 amplifies inflammation and is a crucial mediator of septic shock. *Nature*.

[10] Tao F, Peng L, Deng L, Shao Y, Deng L, Yao H (2013). Association of serum myeloid cells of soluble triggering receptor-1 level with myocardial dysfunction in patients with severe sepsis. *Mediators of Inflammation*.

[11] Gibot S, Kolopp-Sarda M-N, Béné M-C (2004). A soluble form of the triggering receptor expressed on myeloid cells-1 modulates the inflammatory response in murine sepsis. *Journal of Experimental Medicine*.

[12] Ha T, Lu C, Liu L (2010). TLR2 ligands attenuate cardiac dysfunction in polymicrobial sepsis via a phosphoinositide 3-kinase-dependent mechanism. *American Journal of Physiology: Heart and Circulatory Physiology*.

[13] Ha T, Hua F, Grant D (2006). Glucan phosphate attenuates cardiac dysfunction and inhibits cardiac MIF expression and apoptosis in septic mice. *American Journal of Physiology: Heart and Circulatory Physiology*.

[14] Baumgarten G, Knuefermann P, Nozaki N, Sivasubramanian N, Mann DL, Vallejo JG (2001). In vivo expression of proinflammatory mediators in the adult heart after endotoxin administration: the role of toll-like receptor-4. *Journal of Infectious Diseases*.

[15] Bozkurt B, Kribbs SB, Clubb FJ (1998). Pathophysiologically relevant concentrations of tumor necrosis factor- *α* promote progressive left ventricular dysfunction and remodeling in rats. *Circulation*.

[16] Parker MM, McCarthy KE, Ognibene FP, Parrillo JE (1990). Right ventricular dysfunction and dilatation, similar to left ventricular changes, characterize the cardiac depression of septic shock in humans. *Chest*.

[17] Ellrodt AG, Riedinger MS, Kimchi A (1985). Left ventricular performance in septic shock: reversible segmental and global abnormalities. *American Heart Journal*.

[18] Wiggers CJ (1947). Myocardial depression in shock. A survey of cardiodynamic studies. *American Heart Journal*.

[19] Balija TM, Lowry SF (2011). Lipopolysaccharide and sepsis-associated myocardial dysfunction. *Current Opinion in Infectious Diseases*.

[20] Dejager L, Libert C (2008). Tumor necrosis factor alpha mediates the lethal hepatotoxic effects of poly(I:C) in d-galactosamine-sensitized mice. *Cytokine*.

[21] Russell DA, Tucker KK, Chinookoswong N, Thompson RC, Kohno T (1995). Combined inhibition of interleukin-1 and tumor necrosis factor in rodent endotoxemia: improved survival and organ function. *Journal of Infectious Diseases*.

[22] Takeyoshi I, Yoshinari D, Kobayashi M, Kurabayashi M, Morishita Y (2005). A dual inhibitor of TNF-*α* and IL-1 mitigates liver and kidney dysfunction and improves survival in rat endotoxemia. *Hepato-Gastroenterology*.

[23] Gibot S, Massin F, Marcou M (2007). TREM-1 promotes survival during septic shock in mice. *European Journal of Immunology*.

[24] Ulevitch RJ, Tobias PS (1995). Receptor-dependent mechanisms of cell stimulation by bacterial endotoxin. *Annual Review of Immunology*.

[25] Modlin RL, Brightbill HD, Godowski PJ (1999). The toll of innate immunity on microbial pathogens. *The New England Journal of Medicine*.

[26] Ulevitch RJ (1999). Endotoxin opens the tollgates to innate immunity. *Nature Medicine*.

[27] Aderem A, Ulevitch RJ (2000). Toll-like receptors in the induction of the innate immune response. *Nature*.

[28] Gibot S (2005). Clinical review: role of triggering receptor expressed on myeloid cells-1 during sepsis. *Critical Care*.

